# The centrality of behavior change in health systems development – Author's response

**DOI:** 10.9745/GHSP-D-14-00005

**Published:** 2014-02-11

**Authors:** James D Shelton

I heartily welcome Naimoli, Parker, and Heiby's endorsement of behavior change interventions,^1^ as well as their elaboration on behavior change best practices. I maintain my view, however, that behavior change is underappreciated: (1) in its crucial role across all the 6 domains of health interventions, and (2) specifically in the way health systems are conceptualized.

I also admire Naimoli and colleagues' enlightened view in seeing elements of behavior in each of the World Health Organization (WHO) health system building blocks. One can indeed thus say behavior change is “everywhere.” But it is all too often true that things can be categorized as everywhere, or “overarching,” yet be easily overlooked and neglected. One example is the area of nutrition. It pervades virtually every aspect of health and indeed, according to a recent review in the *Lancet*, accounts for a full 45% of child mortality.^2^ Yet partly because it lacks specific focus, emphasis, and funding in global health, it is considerably neglected.

Actually, my concerns about the WHO health system building blocks extend beyond the lack of definitive inclusion of behavior change, to the way health systems under any framework are often conceived. I worry that, all too often, thinking around health systems centers on medical/clinical and often curative services. This should not be the case, of course, since most of the determinants of health relate to lifestyle, and many crucial health services can be provided in the community. So the health system should be broadly defined but still focus on specific, effective interventions. In my experience, the elements I often find get short shrift in discussions of health systems are: behavior change, community-based services, private-sector service delivery, structural/policy approaches such as safe air, water, and roadways and tobacco taxation, and, ironically, even bricks and mortar infrastructure.

I appreciate that Naimoli and colleagues direct us to alternative and arguably more appropriate health system formulations. It is interesting, however, that while the paper they cite by Shakarishvili et al.^3^ does indeed add one additional building block in its composite health system formulation, that additional building block is simply “demand creation.” That addition, of course, only captures 1 of the 6 domains of behavior change. Moreover, it is closely related to clinical/medical service delivery and actually illustrates my concern about the medical/clinical orientation of most thinking about health systems.

I remain concerned that the WHO building-block model remains the predominant health systems conceptual model. One recent example is the landmark Global Health 2035 report from the *Lancet* Commission, summarized in a mere 58 pages in the *Lancet*.^4^ In their section on health systems, they build their analysis around the WHO building blocks. Notably, behavior change gets little attention or credence, although to their credit, the authors do include community-based and structural approaches. I hope the way we think of health systems continues to evolve and to prioritize most explicitly those interventions that have the most impact on health.

## References

[1] Naimoli JF Parker KA Heiby J The centrality of behavior change in health systems development. Glob Health Sci Pract. 2014;2(1): 132–133 10.9745/GHSP-D-13-00170 PMC4168612 25276569

[2] Black RE Victora CG Walker SP Bhutta ZA Christian P de Onis M Maternal and child undernutrition and overweight in low-income and middle-income countries. Lancet. 2013;382(9890): 427–451 10.1016/S0140-6736(13)60937-X 23746772

[3] Shakarishvili G Atun R Berman P Hsiao W Burgess C Lansang MA Converging health systems frameworks: towards a concepts-to-actions roadmap for health systems strengthening in low and middle income countries. Glob Health Gov. 2010;3(2): 1–17 Available from: http://ghgj.org/Shakarishvili_Converging%20Health%20Systems%20Frameworks.pdf22506090

[4] Jamison DT Summers LH Alleyne G Arrow KJ Berkley S Binagwaho A Global health 2035: a world converging within a generation. Lancet. 2013;382(9908): 1898–1955 10.1016/S0140-6736(13)62105-4 24309475

